# Deceptive Intentions: Can Cues to Deception Be Measured before a Lie Is Even Stated?

**DOI:** 10.1371/journal.pone.0125237

**Published:** 2015-05-27

**Authors:** Sabine Ströfer, Matthijs L. Noordzij, Elze G. Ufkes, Ellen Giebels

**Affiliations:** 1 Department of Psychology of Conflict, Risk & Safety, University of Twente, Enschede, The Netherlands; 2 Department of Cognitive Psychology and Ergonomics, University of Twente, Enschede, The Netherlands; Institute of Psychology, GERMANY

## Abstract

Can deceitful intentions be discriminated from truthful ones? Previous work consistently demonstrated that deceiving others is accompanied by nervousness/stress and cognitive load. Both are related to increased sympathetic nervous system (SNS) activity. We hypothesized that SNS activity already rises during intentions to lie and, consequently, cues to deception can be detected before stating an actual lie. In two experiments, controlling for prospective memory, we monitored SNS activity during lying, truth telling, and truth telling with the aim of lying at a later instance. Electrodermal activity (EDA) was used as an indicator of SNS. EDA was highest during lying, and compared to the truth condition, EDA was also raised during the intention to deceive. Moreover, the switch from truth telling toward lying in the intention condition evoked higher EDA than switching toward non-deception related tasks in the lie or truth condition. These results provide first empirical evidence that increased SNS activity related to deception can be monitored before a lie is stated. This implies that cues to deception are already present during the mere intention to lie.

## Introduction

Most of the research on physiological detection of deception has focused on the act of lying, and contrasts specific statements that are either truths or lies [1,2]. These studies build on the fact that there are consistent differences in the physiological response of the sympathetic nervous system when lying or when telling the truth [2–4]. However, categorizing statements as either truths or lies neglects that deception may take a variety of forms, ranging from its most direct form, fabrication, to more subtle forms including half-truths, vagueness, equivocations, and concealments [5]. This implies that deception and lying, although used interchangeably, reflect essentially different constructs. Deception refers to “[…] a deliberate attempt to create in another a belief which the communicator considers to be untrue” [6]. A lie is defined as intentionally making a false statement [7]. Thus, whereas deception refers to a process, lying refers to a specific strategy that can be used during this process. This is an important notion because deceivers tend to stick to the truth as much as possible [8] and to only lie on crucial aspects [9,10].

Theory and empirical data emphasize that lying is cognitively and emotionally taxing [11]. Previous work demonstrated, for instance, that brain areas associated with cognitive processes such as working memory and executive control are more active when lying compared to telling the truth [12–15]. Also, feelings of nervousness and stress may accompany lying [16,17], which can be related to different emotions [18]. These differences are reflected in increased sympathetic nervous system (SNS) activity [2–4].

The aim of the current research is twofold: First we examine whether SNS activity, associated with deception, already can be observed in stages wherein one is telling the truth but does has the intention to lie at a later moment. Second, we examine how SNS activity is affected by moving from a mere intention to deceive to a stage wherein one—in order to keep up their deceptive intentions—actually will be required to lie.

### Truth telling with the intention to lie: Can SNS activity, related to deception, be detected before a lie is stated?

Previous work consistently demonstrated that lying is cognitively and emotionally taxing [19]. Research shows that brain areas associated with cognitive processes such as self- and other monitoring, working memory, and executive control, are more active when lying compared to telling the truth [12–15]. Also, feelings of nervousness and stress frequently accompany lying [16,17]. Based on earlier research [2,4,20–23], we therefore expect SNS activity to be higher during lying compared to truth telling (*Hypothesis 1*).

Moreover, SNS activity may already rise *before* lying, but when having the intention to deceive. Intention refers to a mental representation of planned actions (in this case the aim to deceive others), based on some amount of reasoning and planning [24]. Therefore, many of the cognitive processes needed for lying may already be active during the intention to deceive. This may, for example, reflect monitoring processes related to concerns to appear consistently honest and not to give away cues to deception when switching from truth telling to lying [25,26]. Also, arguably, people may already feel nervous and stressed in the foresight of lying and not just at the moment of lying itself.

Some first evidence that the intention to deceive can be measured, can be found in studies which have tried to separate the ‘act’ of deception from the ‘intention’ to deceive by using a delay in subjects’ answers [22,27–29]. These studies monitored SNS activity while people gave honest and deceptive answers to questions. SNS activity was increased during deceptive answers compared to truthful ones. Even more importantly, SNS activity already increased in the time interval between the question and the answer. In the short moment between question and lying, arousal thus already increased when anticipating lying.

This previous work, however, focused on a single snapshot of the specific moment that people are lying. The current research goes above and beyond these findings by approaching deception as an ongoing process. To do this we make a distinction between the *anticipation* and *action* stage during the process of deception. That is, deceptive interactions for a part exist of an *anticipation* stage in which one has the intention to deceive others but is telling the truth because the content of the interaction does not require lying yet. Only a specific part of the interactions exist of an *action* stage involving interactions that require straightforward lies in order to deceive the other. We predict that increased SNS activity due to the intention to deceive already can be detected in such *anticipation* stages wherein one is telling the truth but has the intention to deceive when necessary *(Hypothesis 2)*.

Moreover, cognitive load and stress caused by preparing to lie should become most taxing when moving to the *action* stage—when the necessity for lying increases. Also, switching between tasks in itself is known to cost effort [30]. Therefore, we assume that the switch from the *anticipation* stage toward the *action* stage especially increases SNS activity *(Hypothesis 3)*.

### The current research

We developed a new paradigm to examine the entire process of deception, contrasting the mere intention to deceive with pure truth telling and lying. Traditionally studies investigating deception contrasted specific truthful statements with lies [2,4,22]. However, such an approach makes it impossible to capture the processes associated with the mere intention to deceive, because intention related processes occur simultaneously with lying processes. In the current work we therefore not just compare SNS activity during lying and truth telling, but also during truth telling with the intention to lie on a later moment.

Moreover, previous studies demonstrated that adding a double task significantly improves the chances of observing cues to deception due to cognitive processes associated with deception [31–34]. A secondary task increases participants’ cognitive demand and impedes the act of deceiving which is also cognitively demanding. This leads to a poorer deception performance and hence more cues to deception [31]. Although studies in the field of deception detection used a variety of double tasks [31–34], studies studying cognitive processes more in general often use arithmetic double tasks [35–37]. In Experiment 1 we used an emotion recognition task, because, while deceiving, people constantly had to read facial expressions to assess whether they were believed or not [38,39]. In Experiment 2 we used a more traditional, arithmetic double task.

In both studies, we measured electrodermal activity (EDA) as an indicator for SNS activity [40,41]. EDA is an index for both stress [42–44] and cognitive load [45–48] (Please note that some studies tried to discriminate stress from cognitive load in EDA [78]. However, usually stress and cognitive load are correlated [79] and difficult tasks (such as deceiving) can induce both [80].). An important advantage of using EDA is that its signal is discriminable, meaning that changes in sympathetic nervous system activity can be detected with one single measurement [41]. Therefore, it is an often-used measure method in field studies with applied relevance [49–52] and the most frequently used physiological measure by scholars and practitioners in the field of deception [53]. An EDA signal measured over time consists of an overall, slow drifting signal, overlaid by short fluctuations, called skin conductance responses [40]. The slow drifting signal is called *tonic* EDA and indicates the overall conductivity of the skin over long time intervals of about ten or more seconds [54]. The overlaying fluctuations are referred to as *phasic* EDA, and are particularly sensitive for short local fluctuations in SNS activity [54,55]. It is therefore likely that tonic EDA will be most suited for comparing longer stages of truth telling—with and without the intention to lie—with those of constantly lying (Hypothesis 1 and 2). Phasic EDA in turn will be especially sensitive to brief changes in arousal, and therefore to measure the switch from *anticipation* toward *action* stage (Hypothesis 3).

## Experiment 1

### Method

#### Participants

Fifty students from a Dutch University participated in the experiment. All subjects provided written informed consent and the Ethics Committee of the Faculty of Behavioral Sciences of the University of Twente approved the experimental protocol. Due to technical failures in measuring skin conductance, three participants were removed, leaving 47 participants for statistical analyses (mean age = 21.01 years, *SD* = 2.28, range = 18–29 years; 21 women).

### Experimental design

#### Blocks

The experiment consisted of three within-subject conditions (lie/truth/intention to lie), and was built up from nine successive blocks (3 lie blocks, 3 truth blocks and 3 intention blocks), presented in a random order. We used three different sequence versions to control for habituation effects. Every block was divided into an *anticipation* and an *action* stage, each consisting of three trials with a question (therefore each block consisted of six trials). In the *action* stage one of the three questions was colored blue (the order of the blue questions was counter-balanced).

In the truth blocks participants were instructed to answer truthfully on all six trials, and in the lie blocks participants had to lie on all six trials. In the *intention blocks*, participants were instructed to answer truthfully on all trials except for the critical trial (with the blue colored question). Because the blue question never occurred in the *anticipation* stage, this stage served as basis to measure truth telling with the intention to lie. The blue question always occurred in one of the final three question trials of the block, hence this stage is referred to as *action* stage.

Important to note is that in the truth and lie blocks participants additionally were instructed to say ‘blue question’ when the blue colored question appeared. This instruction created an expectation regarding the blue questions in all blocks, not just the intention blocks. Electrodermal activity (EDA) differences between the *intention*, *lie* and *truth blocks* therefore are not simply due to *anticipation* or occurrence of a neutral event (the blue question), but can be attributed to the *anticipation* of lying. The trial position of the blue question was unknown to the participant.

#### Trials

**Fig 1:**
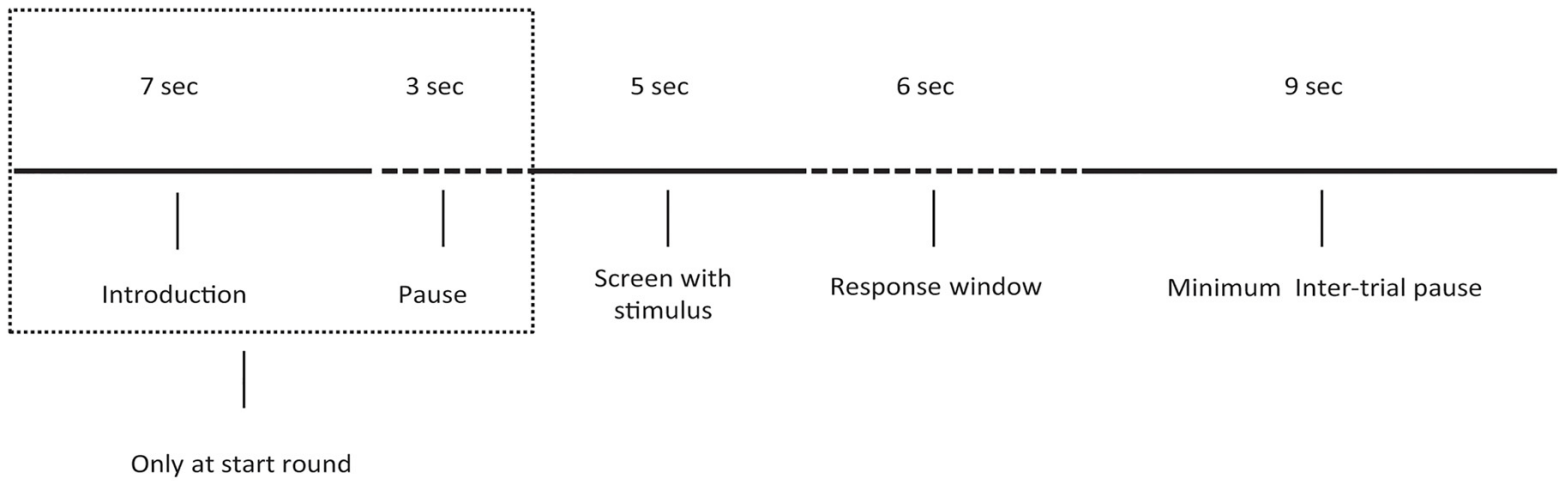
Trial structure. Every trial lasted for 20 seconds. During the first 5 seconds (Screen with stimulus) three objects were shown: a face, a Yes/No question about a face-characteristic and a double task. Then a response window was shown for 6 seconds. In these seconds participants first answered the question and then solved the double task, both orally. Finally, a white screen appeared for 9 seconds before a new trial started.

Participants first saw the stimulus window for 5 seconds, containing a face, a related Yes/No question to that face and a double task. The face stimuli were selected from the Radboud Faces Database [56]. In order to increase external validity, we randomly used faces from males and females, Caucasian and non-Caucasians, as well as from adults and children. The question always referred to a face characteristic (e.g., “Does this person have blue eyes?”). Next, the answer window appeared for 6 seconds. During these 6 seconds participants were asked first to give a full verbal answer to the question (which could be a lie or truth, depending on the block) and then to give the solution to the double task. After the answer window, a white screen appeared for 9 seconds before a new trial began. A trial included a stimulus, a response and a pause (see Fig 1).

#### Double task

**Fig 2:**
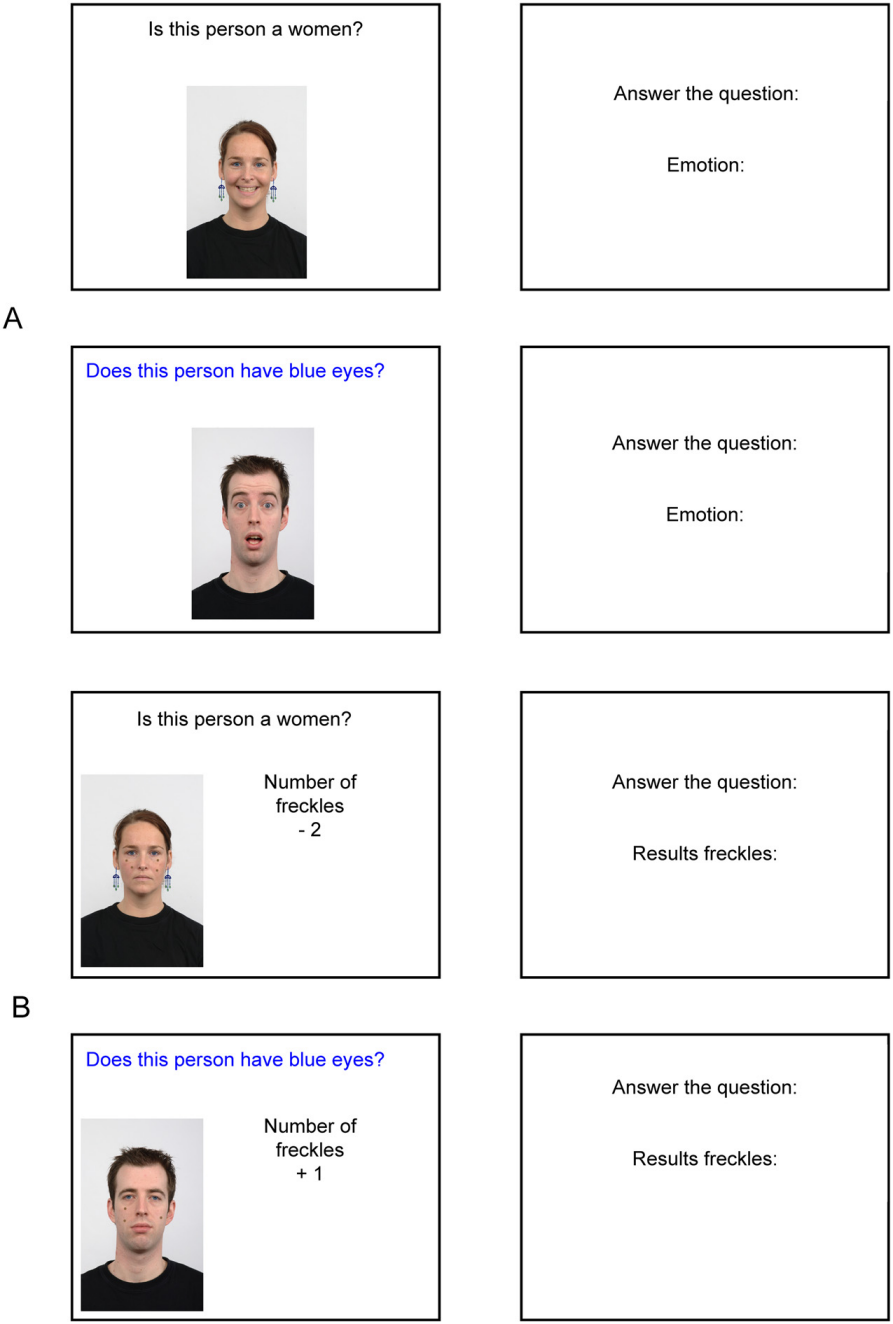
Double task example. Panel A shows an excerpt from Experiment 1 and panel B shows an excerpt from Experiment 2. Both contain two trials, consisting respectively of a question window (duration: 5 seconds) and answer window (duration: 6 seconds). At total each block consisted of 6 such successive trials from which one is colored blue. Whether participants responded with lying or saying ‘blue question’ on the blue question depended on the condition (lying, truth, intention). The two experiments differed with respect to their double task. The double task in Experiment 1 consisted of an emotion recognition task. In the excerpt from panel A participants were expected to say aloud ‘Happy’ and ‘Surprised’, after having answered the question (e.g., ‘Yes, this person is a woman. Happy’). The double task in Experiment 2 consisted of an arithmetic task. In the excerpt from panel B, participants were expected to say aloud ‘2’ and ‘4’ after having answered the question in the answer window (e.g., ‘Yes, this person is a woman. Two’).

Monitoring others’ emotional expressions is an important aspect during deceiving in order to assess whether one is believed or not [38,39]. We constructed the task as such that the face stimuli expressed one of the most recognized emotions across cultures: anger, fear, sadness, disgust, happiness, contempt or surprise [57]. In six blocks, an emotion occurred only once. However, to prevent learning effects, in two blocks one emotion occurred twice and in one block one emotion occurred three times. Across all 54 trials, sadness occurred nine times, happiness and anger eight times, fear six times, disgust eleven times and contempt four times. Participants were instructed to say aloud the emotion that they saw. An example of these trials can be found in Fig 2. In half of the blocks, the facial expression to be recognized was morphed with the neutral face expression of the same face (The original design also contained a between-subject factor, task difficulty. However, analyses with this variable did not render any significant main or interaction effects. We suspect that the difference between the conditions was not strong enough and we therefore eliminated this factor from further analyses.).

#### Procedure

Participants were instructed to answer blocks of six questions. We informed them that with each question a face would be shown to which the questions would be related, for instance a question about hair color. We told them that preceding each block they would get the instruction to lie, tell the truth on all questions, or just to lie on the blue colored question. We informed them that in case they got the instruction to tell the truth or lie on all questions, they had to say aloud ‘blue question’ as soon as they encountered a blue question. We also instructed them to say aloud the emotion they recognized in that face after they had answered the question.

Participants completed one practice block consisting of six trials in the presence of the experimenter who ensured that participants followed the instructions. Then the experimenter left the room and the experiment started. Participants received no feedback about their performance, neither on the double tasks, nor on truth telling and lying. The experiment lasted 26 minutes. To increase the participants’ motivation and involvement in the task, we awarded a prize money of 100 € and 50 € respectively to the ‘best and second best liar’. We also told participants that an expert in lie detection observing their responses via a webcam (a camera was placed next to the monitor so it was visible for the participants) would make this judgment (In reality, the money was allotted among the participants after the experiment).

.

### Measures

#### Electrodermal activity as indicator for sympathetic nervous system activity

Using EDA has several advantages over other physiological measures: It directly reflects activity of the sympathetic nervous system (SNS) without being affected by parasympathetic activity [40,58]. Its signal is distinctive, can be measured unobtrusively and can be detected after one measurement [41]. Because we were interested in long lasting processes (monitoring and preparing) as well as short lasting events (the switching between a stage of truth telling and a stage where a lie takes place), we chose to base our analyses on tonic as well as phasic EDA.

#### Tonic versus Phasic EDA

In EDA research, differences are made between tonic and phasic EDA, which both can be extracted from the raw EDA data [40,55]. EDA measured over a period of time consists of an overall and relatively slow drifting signal on which there are short fluctuations, called skin conductance responses (SCRs) [55,59,60]. The slow drifting signal is called *tonic* EDA and indicates a more general level of arousal over a longer time interval [54]. The SCRs are sensitive for short phasic localized fluctuations in arousal [40].

#### Tonic EDA

Tonic EDA is modulated by chronic stimuli over a longer time interval [41]. Traditionally there are two ways of computing tonic EDA. First, one can average all measurements points distributed across the time window of interest, leaving out the measurement points during the SCRs [40]. By omitting the SCRs, a stable and slowly adapting/changing signal is left, not distorted by spontaneous events [55]. Another method is to measure characteristics of the SCRs observed in the time window of interest, for example the frequency or total amplitude of the SCRs [55]. SCRs reflect the higher-frequency variability of the entire signal [54].

Because we are interested in SNS activity during longer time periods of truth telling and lying, tonic EDA is suitable for comparisons between the three conditions. Tonic EDA increases at any task performance. Even more important, the *anticipation* alone of any task will increase tonic EDA [41]. So, it can measure differences (if present) between truth telling with and without a deceptive intent, which do not differ in task (truth telling) but in *anticipation* (either lying or not on an upcoming question).

#### Phasic EDA

Since phasic EDA is based on SCRs, it is more sensitive to the abrupt, short-lived changes. This makes it a good candidate in our experiments to measure the switch from an *anticipation* stage (e.g., truth telling) to an *action* stage (where e.g., a lie takes place) within a condition.

#### Computing tonic EDA and phasic EDA

Establishing tonic and phasic EDA from one continuous EDA signal often bears difficulties. One of these difficulties is that SCRs often overlap and therefore lose their typical form of a sharp phasic peak. That makes it difficult to tear apart the slow changing tonic EDA from its overlaying SCRs [40]. We used a method by Benedek and Kaernbach [60] which controls for this problem, called the Continuous Decomposition Analysis (CDA). It returns the raw EDA data into a continuous tonic and phasic signal (the overlaying SCRs). Therefore a multi-step deconvolution approach is applied, based upon a physiological model of the SCR shape. The continuous phasic signal is expressed as time integrated area under the SCR and measured in μS*s. The continuous tonic signal is measured in μS.

Because of the slow fluctuations, tonic EDA is suitable for comparisons between the three conditions. Since phasic EDA is based on SCRs, it is more sensitive to the abrupt, short-lived changes when for example switching from anticipation (e.g., truth telling) to acting (e.g., lying) within a condition. Both hypotheses were tested on tonic and phasic EDA. Based on the nature of these two signals we expect to find a stronger pattern of effects for Hypotheses 1 and 2 (overall patterns) in tonic EDA and for Hypothesis 3 in phasic EDA [41].

#### Recording EDA

EDA was measured exodermal (constant voltage) using skin conductance sensors (Thought Technology Ltd., Montreal West, Quebec, Canada), attached to the distal phalanx of the right index and ring fingers [55]. The signal was amplified and recorded using a ProCompInifiniti system (Thought Technology Ltd.). Continuous Decomposition analysis was executed using MATLAB custom code from Ledalab [60].

#### EDA Parameter settings

EDA was recorded at 256 Hz and down-sampled to 16 Hz (well above the 10 Hz after which increases in sample frequency do not significantly alter the EDA parameters) (Benedek and Kaernbach [60] proposed that EDA does not change above a sample rate of 16 Hz. We verified their statement by analyzing the data with 64 Hz. Our results however remained the same.). Parameters were computed with continuous decomposition analysis (CDA). We used a minimum amplitude threshold criterion of .01 μS [55] and iterated the parameter optimization three times. The skin conductance was separated into a continuous tonic and phasic signal, each containing 54 trials per subject.

#### EDA Time window

**Fig 3:**
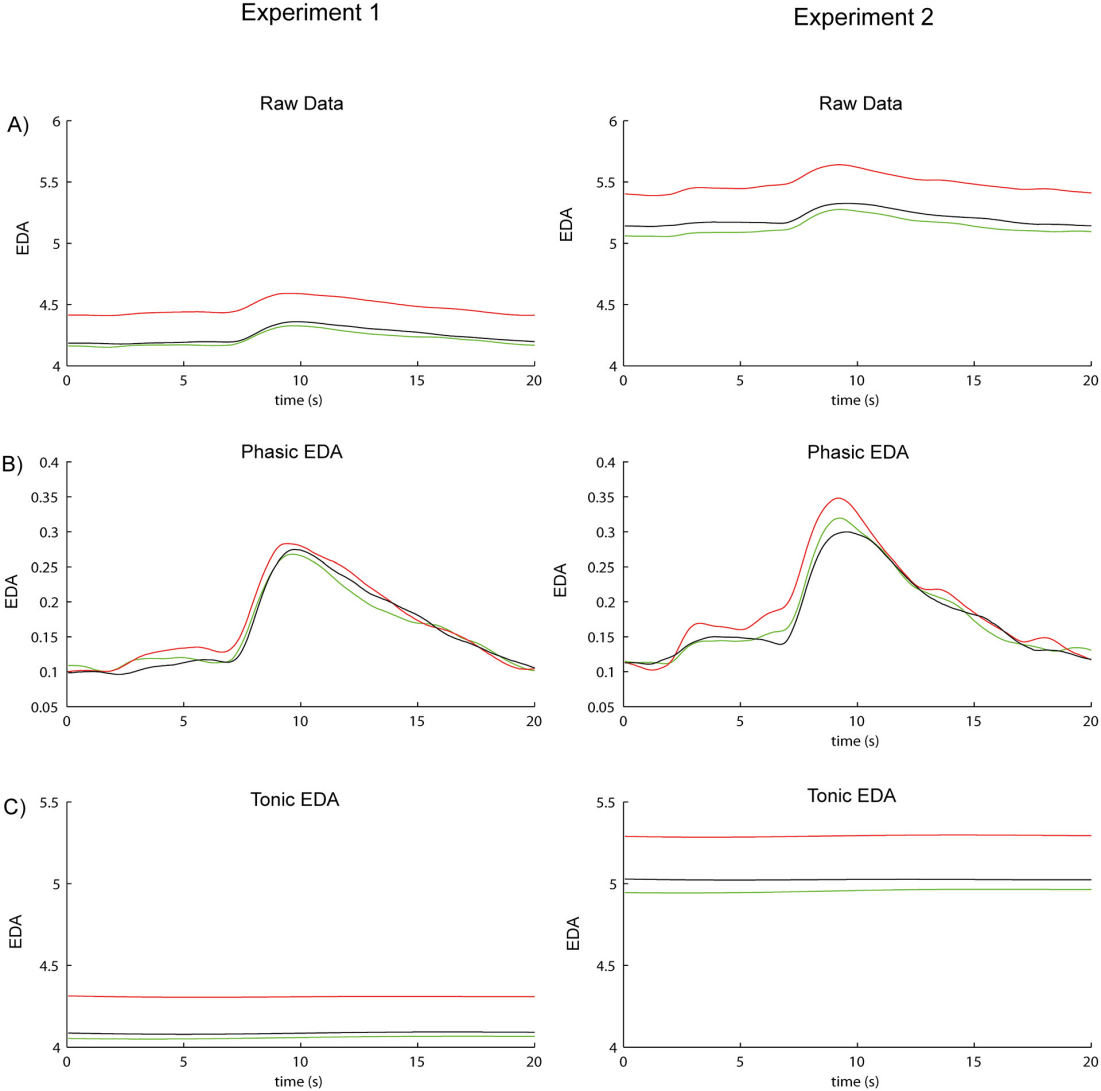
Mean EDA during a trial. We separated raw skin conductance data (A) into a continuous signal of phasic EDA (B) and tonic EDA (C). The graphs present the mean course of skin conductance during a trial (20 s) per condition. The left panel shows EDA for Experiment 1 and the right panel shows EDA for Experiment 2. Both show the same course pattern. For the statistical analysis we used an interval from 2 till 13 seconds (dotted lines). To separate the raw signal into its components, continuous decomposition analysis (CDA) by Benedek and Kaernbach (2010) was used. It is important to note, that phasic EDA is mapped in μS. However, the phasic EDA was time integrated in a later stadium of the CDA analysis. This means that the unit finally changes into μS*s.

SCRs usually have a delay between one and four seconds [55]. Fig 3 shows the mean course of raw EDA, phasic EDA and tonic EDA during a trial (20s) per condition. As can be seen, skin conductance rises about two seconds after stimulus onset. Therefore, we chose a time window ranging from 2 till 13 seconds after stimulus onset (the question), encompassing the moment the question is presented and the moment the answer has to be given. Our statistical analyses based on tonic EDA (μS) and phasic EDA (μS*s) in this time window. As recommended by Boucsein [55], EDA was normalized by taking the natural logarithm. Statistical analyses were performed on log-transformed data, but the reported descriptive statistics were based on the raw data (phasic EDA in μS*s; tonic EDA in μS). The data on which our statistical analyses were performed can be found in S1 Dataset (Experiment 1) and S2 Dataset (Experiment 2).

### Dependent Variables

In deception-detection paradigms it has been established that in a set of trials, the first trial results in atypical EDA fluctuations (e.g., the Guilty knowledge test; [53]). Therefore, we removed the data of the first question trial in a block, leaving two question trials in the *anticipation* stage and three question trials in the *action* stage. We tested Hypotheses 1 and 2 on basis of the *anticipation* stage. The switch from the *anticipation* stage toward the *action* stage enabled us to examine the switch from a stage of truth telling with the intention to lie, toward a stage where a lie occurs. We let the blue question occur on a random trial position within the *action* stage to control for learning effects. Therefore, when contrasting the switch from the *anticipation* toward the *action* stage, we do not refer to the direct switch to a single trial with the blue question but to the switch to the stage that included the blue question. For the statistical analyses we report partial eta square (η_p_^2^) as effect size measure. This effect size measure usually is used in ANOVAs and explains the proportion of variance of an effect that is not explained by other variables in the analysis [61].

### Results

#### Cognitive load during the intention to lie

Our first aim was to compare truth telling with the intention to lie with ‘honest’ truth telling and lying on tonic EDA. Therefore we compared the *anticipation* stage, which spanned the time before the blue question occurred, between conditions. To test our hypotheses we conducted a repeated measures analysis with within-subject factor condition (intention to lie/lie/truth) on tonic EDA (which should be especially sensitive for longer term changes in SNS activity) of the *anticipation* stage. In addition, we also report the results for the phasic EDA.

Supporting our prediction, we found a main effect of condition on tonic EDA for the *anticipation* stage, *F*(2, 92) = 24.95, *p* < .001, η_p_^2^ = .35. Simple effect analyses revealed that EDA in the lie condition (*M* = 4.30, *SE* = .41) was significantly higher compared to the intention (*M* = 4.07, *SE* = .39), *t*(46) = 5.85, *p* < .001, and truth condition (*M* = 4.04, *SE* = .39), *t*(46) = 5.36, *p* < .001. The difference between truth telling with and without the intention to lie was not significant, *t*(46) = 0.85, *p* = .399. We repeated the same ANOVA analysis for phasic EDA within the *anticipation* stage and found no main effect of condition, *F*(2, 92) = 0.66, *p* = .518, η_p_^2^ = .01.

These results supported Hypothesis 1: as expected, lying evoked higher tonic EDA than truth telling. Hypothesis 2, however, was not supported and EDA levels in the intention condition were comparable to the levels in the truth-telling condition. This implies that compared to straightforward truth telling, participants’ SNS activity was not higher when telling the truth while preparing for lying. Lastly, we did not find reliable differences over conditions for phasic EDA levels. This is in accordance with the previously explained idea that phasic EDA is more sensitive to short-lived changes compared to ongoing processes.

#### Cognitive load during the switch from the intention to lie toward lying

Our third prediction was that phasic EDA shows the largest increase from *anticipating* toward *action* stage in the intention condition. Here, participants switch from truth telling with the intention to lie toward a stage where a lie occurs. To rule out that this effect can be attributed to prospective memory (that is, remembering to do something at a particular time [62]), we compared the switch from the *anticipation* toward the *action* stage between the three conditions. We conceptualized the rise in cognitive load as EDA difference between the *anticipation* and *action* stage. In line with this, we subtracted EDA activity of the *anticipation* stage from the *action* stage within all conditions. We conducted a repeated measures ANOVA using as within-subject factor EDA rise (Intention _diff_, Lie _diff_, Truth _diff_) on phasic and tonic EDA. However, the effect of EDA rise on phasic EDA, *F*(2, 92) = 1.87, *p* = .159, η_p_^2^ = .04, nor on tonic EDA, *F*(2, 92) = 1.39, *p* = .255, η_p_^2^ = .03, were significant. The increase of phasic EDA thus did not differ between conditions and Hypothesis 3 therefore was not supported.

### Discussion

Using a new paradigm, we replicated earlier findings, demonstrating that lying evokes higher EDA than truth telling [2,4,20–23]. We found this effect on tonic EDA by comparing stages of constant lying with those of constant truth telling. This is in line with the notion that tonic EDA captures arousal over a long time interval whereas phasic EDA is sensitive to short localized fluctuations in SNS activity. However, we could not confirm our expectation that truth telling with the intention to lie evokes higher EDA than sincere truth telling. Also, the switch between the *anticipation* and *action* stage was not different for the three conditions.

The first aim of Experiment 2 was to replicate Experiment 1, using a different double task. Dual-task interference appears when there is not enough cognitive capacity to process a secondary task on top of the primary task [63]—in our case lying and having the intention to lie. Recognizing expressions plays a role during deception [38,39], and hence an emotion recognition task could interfere with deceptive intent. However, in Experiment 1 we found no difference between the truth and intention conditions. For Experiment 2 we therefore chose a traditional arithmetic double task that has proven empirical ability to exacerbate (subtle) differences in cognitive processing demands of various experimental conditions (e.g., [35–37]). Instead of recognizing an emotion, participants had to solve a sum now. Except for the double task, the Method of Experiment 2 was precisely the same as that of Experiment 1.

## Experiment 2

### Method

#### Participants

Forty-seven new students from a Dutch University participated in the experiment. All subjects provided written informed consent and the experimental protocol was approved by the Ethics Committee of the Faculty of Behavioral Sciences of the University of Twente. Due to technical failures in measuring skin conductance, one participant was removed, leaving 46 participants for statistical analyses (mean age = 20.88 years, *SD* = 1.82, range = 18 through 27; 25 women).

#### Double task

For the arithmetic double tasks we chose the same face stimuli as in the emotion recognition task [56]. However, this time the face stimuli had a neutral expression with ‘printed’ dots on it (range: 1 to 6 dots). The printed dots served as basis for the ‘arithmetic’ double task. In half of the blocks, these dots had to be added or subtracted with a number between one and six and in the other half of the blocks to be multiplied with a number between seven and 27 (see Fig 2). Again, each time the correct answer had to be said out loud. Participants received no feedback about whether they solved the sum correctly.

#### Procedure

The same procedure as in Experiment 1was applied with only one difference: The instruction about the double task. Participants completed one practice block consisting of six trials in the presence of the experimenter. We informed participants that the faces contained a number of “freckles”. On basis of these freckles they would have to solve a sum, for instance “Number of freckles plus two”. We instructed participants to say aloud the answer to the sum after they answered the question about the face.

### Results

#### Cognitive load during the intention to lie

An ANOVA with within-subject factor condition (intention to lie/lie/truth), showed, as in Experiment 1, a main effect of condition on tonic EDA for the *anticipation* stage, *F*(2, 90) = 18.53, *p* < .001, η_p_^2^ = .29. Simple effect analyses revealed that EDA in the lie condition (*M* = 5.28, *SE* = .60) was significantly higher compared to the intention (*M* = 5.01, *SE* = .55), *t*(45) = 3.73, *p* = .001 and truth condition (*M* = 4.93, *SE* = .55), *t*(45) = 5.09, *p* < .001. Moreover, different from the results of Experiment 1, but in line with Hypothesis 2 the results showed that, compared to the truth condition, EDA in the intention condition was significantly higher, *t*(45) = 2.69, *p* = .010.

We repeated the ANOVA for phasic EDA within the *anticipation* stage and again found a main effect of condition, *F*(2, 90) = 3.60, *p* = .031, η_p_^2^ = .07. Simple effect analyses revealed that EDA was significantly higher in the lie (*M* = 2.98, *SE* = .40), compared to the intention condition (*M* = 2.61, *SE* = .35), *t*(45) = 2.37, *p* = .022, but not compared to truth condition (*M* = 2.80, *SE* = .38), *t* (45) = 1.31, *p* = .196. The difference of EDA between the intention and truth condition was not significant, *t*(45) = 1.64, *p* = .107.

In line with Hypothesis 1, and the results of Experiment 1, we found that tonic EDA was highest during lying. Moreover the results of Experiment 2 provided support for Hypothesis 2 as well. Compared to the truth telling condition, EDA was already elevated when participants had the intention to deceive, but were not required to lie yet. As expected, this difference was found when using the tonic but not the phasic EDA-data.

#### Cognitive load during the switch from the intention to lie toward lying

**Fig 4:**
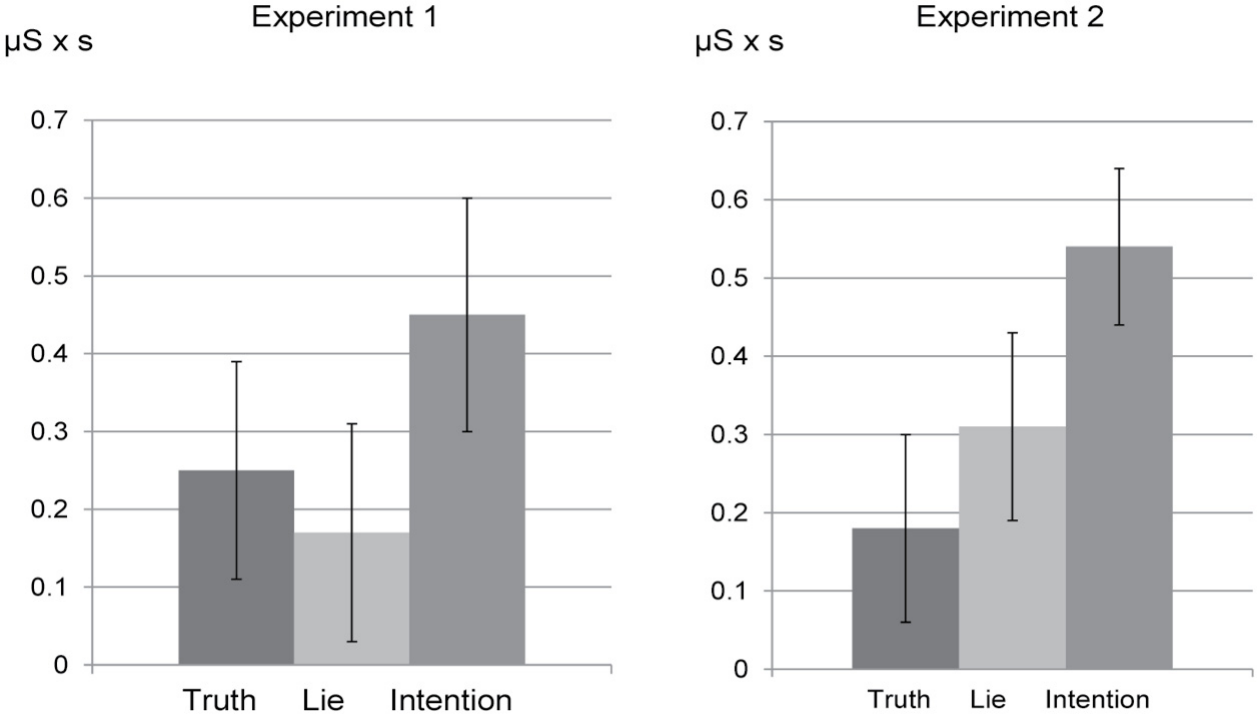
The switch from the anticipation to the action stage. The figure displays the switch from the *anticipation* to the *action* stage. Experiment 1: Mean rise in phasic EDA (with standard errors within parentheses) for the truth, lie and intention condition were 0.25 (0.14), 0.17 (0.14), 0.45 (0.15) respectively. Experiment 2: Mean rise in phasic EDA (with standard errors within parentheses) for the truth, lie and intention condition were 0.18 (0.12), 0.31 (0.12), 0.54 (0.10) respectively.

Similar to Experiment 1, we conducted a repeated measures ANOVA using as within-subject factor EDA rise (Intention _diff_, Lie _diff_, Truth _diff_) on phasic and tonic EDA to compare the switch from the *anticipation* toward the *action* stage between the three conditions. We found a main effect of EDA rise for phasic EDA, *F*(2, 90) = 4.22, *p* = .018, η_p_^2^ = .09 (see Fig 4). In support of Hypothesis 3, simple effect analyses following the ANOVA revealed that the EDA rise within the intention condition (*M* = 0.54, *SE* = .10) was significantly higher compared to that in the lie condition (*M* = 0.31, *SE* = .12), *t*(45) = 2.13, *p* = .038, and truth condition (*M* = 0.18, *SE* = .12), *t*(45) = 2.50, *p* = .016. EDA rise did not differ between the lie and truth condition, *t*(45) = 1.21, *p* = .232.

For tonic EDA we found a marginal significant main effect of EDA rise, *F*(2, 90) = 3.06, *p* = .051, η_p_^2^ = .06. Simple-effect analyses revealed that the rise was highest in the intention to lie condition (*M* = 0.07, *SE* = .02), and that this significantly differed from the rise in the lie condition (*M* = .03, *SE* = .01), *t*(45) = 2.45, *p* = .018, but not in the truth condition (*M* = .07, *SE* = .02), *t*(45) = 0.13, *p* = .896. EDA rise did not significantly differ between the lie and truth condition, *t*(45) = 1.88, *p* = .066.

We could support Hypothesis 3 for Experiment 2: The switch from *anticipation* toward *action* stage on phasic EDA was highest in the intention condition. For these results the patterns of the phasic EDA were clearer than those of the tonic EDA. This is in line with the notion that phasic EDA is sensitive for brief changes in SNS activity [54,55].

## General Discussion

While over the past decades studies have been comparing lying and truth telling to detect cues to deception [26], our study is the first to find evidence that deceptive intent alone can contain cues in terms of increased SNS activity. In two experiments we tested whether the intention to deceive can be discriminated in terms of SNS activity from both truthful as well as entirely deceptive accounts. Using skin conductance (EDA) as an indicator for SNS activity, our results demonstrate that psychophysiological measures contain markers that may discriminate honest and deceitful intentions even before a lie is stated.

Similarly to previous studies we have found higher SNS activity during lying than truth telling [2,4,20–23]. In reality deceivers often alternate between truth telling and lying in order to present a believable story, and stick to the truth most of the time [8–10]. This implies that a great part of deceptive attempts exist of truth telling with the intention to lie. The current study complements the field of deception detection by moving one step further than simply contrasting lies with truth, and showing that truth telling with the intention to deceive in itself may evoke higher SNS activity than ‘sincere’ truth telling. These findings are in line with earlier studies, which have shown that SNS activity already increased in the short interval between a question and given lie [22,27–29]. We, in contrast, focused on the intention to deceive *before* the need for a specific lie emerges. By doing so we were able to study deception as an ongoing process and demonstrate that lying is a crucial, but short element of the deception process. Furthermore, we found that particularly the switch from truth telling with deceptive intention toward an action stage involving a straightforward lie evokes higher EDA than switching toward an action stage requiring giving a non-deceptive statement.

We do have to note that the results from Experiment 1 and Experiment 2 differed to some extent. Specifically, the prediction that truth telling with the intention to deceive increases EDA compared to sincere truth telling was supported by the results of Experiment 2 but not by Experiment 1. Interestingly, EDA differences between the conditions seemed to become more in line with our predictions when using an arithmetic double task compared to an emotion recognition double task, especially with regard to truth telling with and without the intention to lie. Because we used these double tasks in two independent studies, we can only speculate why the arithmetic double task may have interfered more with the intention to lie and hence, increased the difference between truth telling with and without the intention to lie. A reason could be that, for instance, the mental operations needed to solve the equations are qualitatively different from emotion recognition. Solving an equation may need mental operations that are stretched in time whereas emotion recognition takes place within a brief moment. Also, an assignment to solve equations may be more concrete than emotion recognition, which could have led participants to focus more on solving the sum than on emotion recognition. Future research could systematically manipulate types of double tasks to further investigate which type of task is best suited to increase the chance of observing cues to deception during the intention to deceive.

It is most likely that the elevated EDA-levels during lying and the intention to lie observed in the present research are indicators for increased arousal, and are caused by stress and cognitive load. There is an abundance of research demonstrating that EDA rises with both stress/nervousness [42,43] and cognitive load [45–48,64]. Some studies found that EDA actually decreases with task difficulty [65,66]. Ikehara and Crosby [65] for instance, compared an easy and difficult task and found, contrary to their expectations, that the latter induced lower EDA. Interestingly they ascribed their findings to the task design and speculate that the easy task was too easy and tedious, and therefore could actually be more stressful than the difficult task. It is therefore likely that in these studies increased EDA was a result of increased emotional arousal rather than decreased cognitive load.

It is well-known that lying requires cognitive processes unique for lying, such as suppressing the truth while making a counterfactual statement [67,68]. However, the current work suggests that SNS activity may already increase during the mere intention to lie. We therefore propose that some cognitive processes relevant to lying, such as self-monitoring and lie preparation, already may be relevant during truth telling with the intention to lie. An important avenue for future research therefore may be to investigate what kind of processes underlie the increase in SNS activity during lying and/or the intention to lie.

Research on the behavioral correlates of deception show that particularly high-stakes lies (often occurring in real-life) are associated with intense emotions [69], while relatively low-stakes lies (in experimental studies such as the current studies) are typically associated with cognitive load [26,69]. On the one hand, because the topic of deception in the present research concerned relatively low-stakes, it may be less likely that strong emotions such as guilt or anxiety were aroused in the current paradigm. On the other hand, because participants were led to believe that a lie expert would judge them and that best two liars would receive a monetary prize, it is likely that deceiving was accompanied by excitement to fool the lie expert. This so called ‘duping delight’ [70] could have resulted in elevated EDA during lying and the mere intention to lie compared to truth telling as a result of emotional arousal as well. It would be interesting to investigate how stakes moderate the type of processes (cognitive versus emotional) causing an increase in EDA.

### Limitations and future research

When further developing the current paradigm, we advise to take into consideration several points. In the present work we decided to remove the first of six trials in a block, leaving two trials in the *anticipation* and three trials in the *action* stage. Herewith we aimed to prevent that atypical fluctuation commonly seen in the first trial of a set trials distorted our data [53]. Importantly, the different lengths of the two stages should not be problematic for the current findings. Both stages have the same lengths across the three conditions and also, we took the average of the trials within the stages. However, for follow-up studies we advise to expand on the number of trials in order to create *anticipation* and *action* stages of equal length. Also through adding more trials, a post action stage could be built in. The latter would be interesting in order to measure the reversed switch from lying toward truth telling.

We further should point out that the design of these studies was not optimized to examine the switch from truth telling toward lying within an attempt to deceive. Originally, the paradigm was designed to measure truth telling with the intention to lie. To be able to compare the anticipating stages for all three conditions, we took trial 1–3 as the *anticipation* stage, because the blue question never occurred before the 4th trial. The blue question occurred on different trials within the action stage in order to prevent that the moment to lie would become predictable for participants. Ideally we would have analyzed the switch from the *anticipation* toward the blue question separately for the blocks on trial level. However, unfortunately our data set was too small to run these analyses while having sufficient statistical power. We therefore took the average of trial 4, 5 and 6 as the action stage. We realize that in some instances the blue question occurred at the very end of this phase. However, because it was clear that each block only had 6 trials, even in these cases participants would already be triggered and ready for action in the fifth trial. We therefore encourage future research to examine the switch from truth telling toward lying on trial level.

Generally, it would be interesting to design follow-up research that replaces the face stimuli with real humans to create a dynamic face-to-face interaction. This could generate more interpersonal monitoring processes, because the deceived person is the person checking one’s credibility. Also, a real person’s facial expression is not static and therefore monitoring a real person’s face may induce more long-lasting interference with lying/the intention to lie than the face recognition of static faces (as this was the case at the emotion-recognition task in Experiment 1).

### Conclusion

The present findings suggest that the current scientific discourse surrounding lie detection might have been approached too dichotomously. First, lie detection research usually differentiates between lying and truth telling, which resonates in well-known deception detection methods. For instance, the Comparison Question Test [71] measures different physiological parameters and compares them on Yes/No answers that could either be a lie or the truth [72]. Second, looking at lies and truths in isolation does insufficiently take into account that deception is a ‘process in which someone tries to create in another a belief which (…) is untrue’ [73]. Creating such a belief can be achieved through different means. Arguably crucial is the intention to deceive someone, which underlies the entire attempt to deceive [10], including lying and truth telling parts. And third, the findings add to an upcoming field in deception detection research: The ability to discover lies about *future* intentions [74]. In line with the dichotomous focus on lying, most research in this new area examines whether a particular statement about the future is truthful or not [75–77]. We would suggest that, also for this field, investigating deception as an ongoing process would provide a valuable alternative approach.

## Supporting Information

S1 Dataset EDA data of Experiment 1. EDA data export from Ledalab, ordered by output variable, block and then trial.
(XLS)

S2 Dataset EDA Data of Experiment 2. EDA data export from Ledalab, ordered by output variable, block and then trial.
(XLS)
